# Supplementary material for: Baseline Blood Levels of Mucin-1 Are Associated with Crucial On-Treatment Adverse Outcomes in Patients with Idiopathic Pulmonary Fibrosis Receiving Antifibrotic Pirfenidone
Source: Biomedicines. 2024 Feb 8;12(2):402. doi: 10.3390/biomedicines12020402 (PMC10886731; doi:10.3390/biomedicines12020402)
Supplement: Supplementary file 1 [file biomedicines-12-00402-s001.zip › biomedicines-2858036-supplementary.pdf]

Baseline blood levels of mucin-1 are associated with crucial on-treatment adverse outcomes in patients with idiopathic pulmonary fibrosis receiving antifibrotic pirfenidone

Tang-Hsiu Huang, Sheng-Huan Wei, Hung-I Kuo, Hsin-Yu Hou, Chin-Wei Kuo, Yau-Lin Tseng, Sheng-Hsiang Lin, Chao-Liang Wu

Supplementary Materials

Corresponding Author:
Chao-Liang Wu, PhD, Professor Emeritus
Address: Department of Biochemistry and Molecular Biology, College of Medicine, National Cheng Kung University,
Cheng-Hsing Campus, No.1, Dasyue Road, East District,
Tainan City 701, Taiwan
Telephone number: +886-6-2353535 ext. 5536 / Fax number: +886-6-2741692
E-mail: wumolbio@mail.ncku.edu.tw

Table of Content

Supplemental Material	Page
**Supplemental Table S1.** Characteristics and outcomes of patients with and without on-treatment acute exacerbation of idiopathic pulmonary fibrosis (AE-IPF) during the follow-up period.	3
**Supplemental Table S2.** Cox proportional-hazards regression and subdistribution hazard regression on the risk of on-treatment acute exacerbation of idiopathic pulmonary fibrosis (AE-IPF) during the follow-up period.	4
**Supplemental Table S3.** Characteristics and outcomes of patients with and without on-treatment severe adverse outcomes (SAO) during the follow-up period.	5
**Supplemental Table S4.** Cox proportional-hazards regression on the risk of on-treatment early severe adverse outcomes (SAO).	7
**Supplemental Table S5.** Cox proportional-hazards regression on the risk of on-treatment severe adverse outcomes (SAO) anytime during the follow-up period.	8
**Supplemental Table S6.** Sensitivity analysis replacing "GAP stages" in all the multi-variable models with its individual constituting variables (sex, age, FVC, D_{LCO}).	9
**Supplemental Figure S1.** Sensitivity analyses of the multi-variable Cox proportional hazards regression models for acute exacerbation of idiopathic pulmonary fibrosis (AE-IPF).	10
**Supplemental Figure S2**. Sensitivity analyses of the multi-variable Cox proportional hazards regression models for early severe adverse outcomes (SAO).	11
**Supplemental Figure S3.** Sensitivity analyses of the multi-variable Cox proportional hazards regression models for severe adverse outcomes (SAO) anytime during the follow-up period.	12
**Appendix S1**. Formulae for the calculation of rates of changes in pulmonary functional parameters.	13

Supplemental Table S1. Characteristics and outcomes of patients with and without on-treatment acute exacerbation of idiopathic pulmonary fibrosis (AE-IPF) during the follow-up period.

Baseline characteristics and outcomes		Without AE-IPF (N = 50)	With AE-IPF (N = 20)	P – value[a]
Age, years		76.1 (± 9.3)	73.1 (± 9.8)	0.253
Sex	Female, n (%)	8 (16)	4 (20)	0.732
	Male, n (%)	42 (84)	16 (80)	
Body mass index, kg/m^2		23.9 (± 3.6)	22.6 (± 3.2)	0.189
Body surface area, m^2		1.66 (± 0.17)	1.64 (± 0.14)	0.711
Cigarette smoking status	Never smoker, n (%)	19 (38)	6 (30)	0.809
	Current smoker, n (%)	5 (10)	2 (10)	
	Former smoker, n (%)	26 (52)	12 (60)	
Charlson comorbidity index		5 (4 – 7)	4 (3 – 6)	0.122
Echocardiographic evidence of pulmonary hypertension, n (%)		25 (50)	10 (50)	1.000
Baseline plasma mucin-1 level, ng/mL		1.34 (0.68 – 2.12)	3.88 (2.52 – 5.96)	< 0.001
Baseline FVC, L		1.99 (± 0.50)	1.97 (± 0.54)	0.964
Baseline FVC, % predicted		66 (± 10)	64 (± 13)	0.482
Baseline D_{LCO}, mmol/min/kPa		3.04 (± 1.21)	1.83 (± 0.76)	< 0.001
Baseline D_{LCO}, % predicted		59 (± 22)	35 (± 13)	< 0.001
Stages based on the GAP index	Stage 1, n (%)	11 (22)	2 (10)	0.035
	Stage 2, n (%)	34 (68)	11 (55)	
	Stage 3, n (%)	5 (10)	7 (35)	
Dosing	Low (1200 mg/day), n (%)	31 (62)	9 (45)	0.285
	High (1800 mg/day), n (%)	19 (38)	11 (55)	
On-treatment mortality, n (%)		12 (24)	18 (90)	< 0.001
On-treatment lung transplantation, n (%)		1 (2)	-	-
Time to on-treatment AE-IPF, weeks		-	14.9 (7.9 – 50.6)	-
Duration of pirfenidone therapy, weeks		65.1 (26.3 – 136.0)	33.6 (14.2 – 59.8)	0.047

Categorical data are presented as counts and percentages, and continuous variables are presented as means (±standard deviation) or medians (interquartile range) if non-normally distributed. AE-IPF, acute exacerbation of idiopathic pulmonary fibrosis; D_{LCO}, diffusion capacity for carbon monoxide; FVC, forced vital capacity; GAP, gender, age, physiology. [a] P-value for the comparison between patients with and those without AE-IPF.

Supplemental Table S2. Cox proportional-hazards regression and subdistribution hazard regression on the risk of on-treatment acute exacerbation of idiopathic pulmonary fibrosis (AE-IPF) during the follow-up period.

Plasma mucin-1 levels (ng/mL)	Crude HR[a] of AE-IPF (95% CI)	*P*-value	Adjusted HR[a] of AE-IPF (95% CI)	*P*-value	Subdistribution HR[b] of AE-IPF (95% CI)	*P*-value
Dichotomous model:						
≥ 2.5 versus < 2.5	10.49 (3.48 – 31.68)	< 0.001	14.07 (4.26 – 46.49)	< 0.001	10.85 (3.76 – 31.28)	< 0.001
Subgroups based on the mucin-1 levels:						
< 1.5	Reference group		Reference group		Reference group	
≥ 1.5 but < 2.5	5.26 (0.54 – 51.20)	0.153	7.85 (0.69 – 89.32)	0.097	8.54 (0.63 – 116.42)	0.110
≥ 2.5 but < 3.5	11.78 (1.31 – 105.74)	0.028	20.42 (2.04 – 204.25)	0.010	15.69 (1.44 – 171.00)	0.024
≥ 3.5	56.06 (6.96 – 451.33)	< 0.001	101.96 (10.43 – 996.47)	< 0.001	70.33 (8.01 – 617.13)	< 0.001

AE-IPF, (on-treatment) acute exacerbation of idiopathic pulmonary fibrosis; HR, hazard ratio; 95% CI, 95% confidence interval.

[a] Derived from single-variate and multi-variable Cox proportional hazard regression analysis; for the multi-variable analysis, the following covariables were incorporated for adjustment: gender-age-physiology (GAP) stages, smoking status, Charlson comorbidity index, echocardiographic evidence of pulmonary hypertension. [b] Derived from subdistribution hazard regression analysis that adjusted GAP stages, smoking status, Charlson comorbidity index, echocardiographic evidence of pulmonary hypertension, and further controlled for the competing risk of on-treatment severe adverse outcomes (SAO, including on-treatment lung transplantation and all-cause mortality).

Supplemental Table S3. Characteristics and outcomes of patients with and without on-treatment severe adverse outcomes (SAO) during the follow-up period.

Baseline characteristics and outcomes		Without SAO (N = 39)	With SAO (N = 31)	P – value[a]
Age, years		74.6 (± 9.5)	76.1 (± 9.6)	0.558
Sex	Female, n (%)	6 (15)	6 (19)	0.754
	Male, n (%)	33 (85)	25 (81)	
Body mass index, kg/m^2		24.0 (± 3.6)	23.0 (± 3.3)	0.287
Body surface area, m^2		1.67 (± 0.17)	1.64 (± 0.15)	0.274
Cigarette smoking status	Never smoker, n (%)	13 (33)	12 (39)	0.656
	Current smoker, n (%)	5 (13)	2 (6)	
	Former smoker, n (%)	21 (54)	17 (55)	
Charlson comorbidity index		5 (4 – 7)	5 (4 – 6)	0.415
Echocardiographic evidence of pulmonary hypertension, n (%)		21 (54)	14 (45)	0.631
Baseline plasma mucin-1 level, ng/mL		0.96 (0.62 – 1.70)	2.98 (1.79 – 4.16)	< 0.001
Patients with SAO < 2 years			3.53 (2.12 – 5.14)	< 0.001
Patients with SAO ≥ 2 year			2.40 (1.53 – 3.10)	0.050
Baseline FVC, L		2.07 (± 0.46)	1.87 (± 0.54)	0.094
Baseline FVC, % prediction		68 (± 10)	63 (± 12)	0.061
Baseline D_{LCO}, mmol/min/kPa		3.02 (± 1.20)	2.33 (± 1.17)	0.027
Baseline D_{LCO}, % prediction		55 (± 21)	49 (± 24)	0.165
Stages based on the GAP index	Stage 1, n (%)	8 (21)	5 (16)	0.229
	Stage 2, n (%)	27 (69)	18 (58)	
	Stage 3, n (%)	4 (10)	8 (26)	
Dosing	Low (1200 mg/day), n (%)	24 (62)	16 (52)	0.470
	High (1800 mg/day), n (%)	15 (38)	15 (48)	
On-treatment AE-IPF, n (%)		2 (5)	18 (58)	< 0.001
On-treatment death, n (%)		-	30 (97)	-
Due to AE-IPF, n (%)		-	12 (39)	-
Due to IPF progression, n (%)		-	8 (27)	-
Due to infectious pneumonia, n (%)		-	5 (16)	-
Due to extra-pulmonary infection, n (%)		-	2 (6)	-
Due to cardiovascular events, n (%)		-	2 (6)	-
Due to extrapulmonary cancer, n (%)		-	1 (3)	-
On-treatment lung transplantation, n (%)		-	1 (3)	-
Time to on-treatment SAO, weeks		-	52.6 (15.0 – 112.6)	-
Duration of pirfenidone therapy, weeks		60.4 (29.4 – 143.1)	55.7 (15.0 – 110.3)	0.277

Categorical data are presented as counts and percentages, and continuous variables are presented as means (±standard deviation) or medians (interquartile range) if non-normally distributed. AE-IPF, acute exacerbation of idiopathic pulmonary fibrosis; D_{LCO}, diffusion capacity for carbon monoxide; FVC, forced vital capacity; GAP, gender, age,

physiology; SAO, severe adverse outcomes (including on-treatment lung transplantation and all-cause mortality).

[a] *P*-value for the comparison between patients with and those without SAO.

Supplemental Table S4. Cox proportional-hazards regression on the risk of on-treatment ***early*** severe adverse outcomes (SAO) within 2 years of pirfenidone treatment.

Plasma mucin-1 levels (ng/mL)	Crude HR[a] of SAO within 2 years (95% CI)	*P*-value	Adjusted HR[a] of SAO within 2 years (95% CI)	*P*-value
Dichotomous model:				
≥ 2.5 versus < 2.5	5.91 (2.30 – 15.18)	< 0.001	7.87 (2.86 – 21.70)	< 0.001
Subgroups based on the mucin-1 levels:				
< 1.5	Reference group		Reference group	
≥ 1.5 but < 2.5	3.93 (0.72 – 21.48)	0.114	3.26 (0.56 – 19.07)	0.190
≥ 2.5 but < 3.5	7.36 (1.43 – 37.95)	0.017	8.25 (1.56 – 43.66)	0.013
≥ 3.5	16.44 (3.61 – 74.84)	< 0.001	26.56 (4.99 – 141.33)	< 0.001

HR, hazard ratio; SAO, severe adverse outcomes (including on-treatment lung transplantation and all-cause mortality); 95% CI, 95% confidence interval.

[a] Derived from single-variate and multi-variable Cox proportional hazard regression analysis; for the multi-variable analysis, the following covariables were incorporated for adjustment: gender-age-physiology (GAP) stages, smoking status, Charlson comorbidity index, and echocardiographic evidence of pulmonary hypertension.

Supplemental Table S5. Cox proportional-hazards regression on the risk of on-treatment severe adverse outcomes (SAO) anytime during the follow-up period.

Plasma mucin-1 levels (ng/mL)	Crude HR[a] of SAO (95% CI)	*P*-value	Adjusted HR[a] of SAO (95% CI)	*P*-value
Dichotomous model:				
≥ 2.5 versus < 2.5	4.07 (1.94 – 8.53)	< 0.001	4.68 (2.11 – 10.39)	< 0.001
Subgroups based on the mucin-1 levels:				
< 1.5	Reference group		Reference group	
≥ 1.5 but < 2.5	2.75 (0.80 – 9.49)	0.109	2.35 (0.65 – 8.51)	0.193
≥ 2.5 but < 3.5	5.33 (1.60 – 17.81)	0.007	6.17 (1.79 – 21.23)	0.004
≥ 3.5	8.53 (2.72 – 26.73)	< 0.001	8.18 (2.43 – 27.55)	< 0.001

HR, hazard ratio; SAO, severe adverse outcomes (including on-treatment lung transplantation and all-cause mortality); 95% CI, 95% confidence interval.

[a] Derived from single-variate and multi-variable Cox proportional hazard regression analysis; for the multi-variable analysis, the following covariables were incorporated for adjustment: gender-age-physiology (GAP) stages, smoking status, Charlson comorbidity index, and echocardiographic evidence of pulmonary hypertension.

Supplemental Table S6. Sensitivity analysis replacing "GAP stages" in all the multi-variable models with its individual constituting variables (sex, age, FVC, D_{LCO}).

**Plasma mucin-1 levels (ng/mL)**	**Adjusted HR[a] for AE-IPF (95% CI)**	***P*-value**	**Subdistribution HR[b] for AE-IPF (95% CI)**	***P*-value**
Dichotomous model:				
≥ 2.5 versus < 2.5	21.84 (3.90 – 122.16)	< 0.001	14.34 (3.03 – 67.96)	< 0.001
Subgroups based on mucin-1 levels:				
< 1.5	Reference group		Reference group	
≥ 1.5 but < 2.5	5.17 (0.37 – 72.42)	0.222	8.07 (0.43 – 149.89)	0.160
≥ 2.5 but < 3.5	48.30 (1.96 – 1191.26)	0.018	52.73 (2.06 – 1346.69)	0.016
≥ 3.5	70.25 (4.48 – 1100.84)	0.002	55.97 (3.58 – 874.42)	0.004

**Plasma mucin-1 levels (ng/mL)**	**Adjusted HR[c] for SAO (95% CI)**	***P*-value**
Dichotomous model:		
≥ 2.5 versus < 2.5	3.47 (1.55 – 7.78)	0.002
Subgroups based on mucin-1 levels:		
< 1.5	Reference group	Reference group
≥ 1.5 but < 2.5	1.55 (0.40 – 5.94)	0.525
≥ 2.5 but < 3.5	4.17 (1.07 – 16.18)	0.039
≥ 3.5	4.75 (1.10 – 20.59)	0.037

**Plasma mucin-1 levels (ng/mL)**	**Adjusted HR[c] for SAO within 2 years (95% CI)**	***P*-value**
Dichotomous model:		
≥ 2.5 versus < 2.5	4.67 (1.65 – 13.24)	0.004
Subgroups based on mucin-1 levels:		
< 1.5	Reference group	Reference group
≥ 1.5 but < 2.5	2.46 (0.39 – 15.76)	0.341
≥ 2.5 but < 3.5	4.40 (0.67 – 29.11)	0.124
≥ 3.5	13.27 (2.08 – 84.85)	0.006

AE-IPF, (on-treatment) acute exacerbation of idiopathic pulmonary fibrosis; HR, hazard ratio; 95% CI, 95% confidence interval. [a] Derived from multi-variable Cox proportional hazard regression analysis incorporating the following covariables: sex, age, forced vital capacity (FVC, in % prediction), diffusion capacity of the lung for carbon monoxide (D_{LCO}, in % prediction), Charlson comorbidity index (CCI), smoking status, and echocardiographic evidence of pulmonary hypertension. [b] Derived from subdistribution hazard regression analysis that adjusted sex, age, FVC (in % prediction), D_{LCO} (in % prediction), CCI, smoking status, and echocardiographic evidence of pulmonary hypertension. [c] Derived from multi-variable Cox proportional hazard regression analysis incorporating the following covariables: sex, age, FVC (in % prediction), D_{LCO} (in % prediction), CCI, smoking status, and echocardiographic evidence of pulmonary hypertension.

Supplemental Figure S1. Sensitivity analyses of the multi-variable Cox proportional hazards regression models for the risk of acute exacerbation of idiopathic pulmonary fibrosis (AE-IPF).

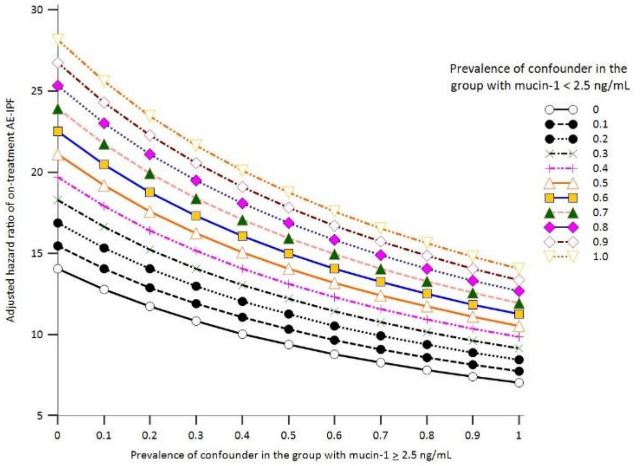

Supplemental Figure S1a

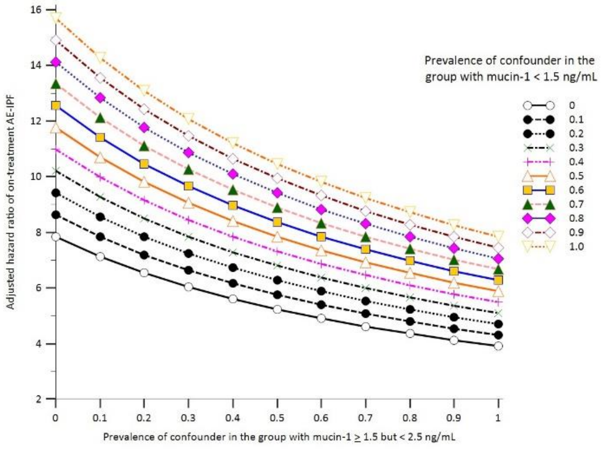

Supplemental Figure S1b

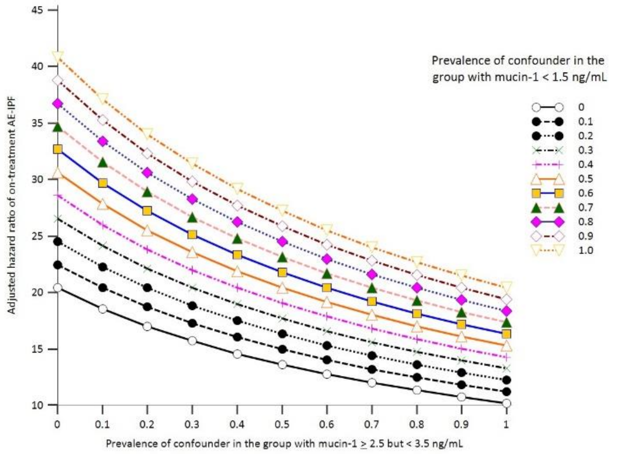

Supplemental Figure S1c

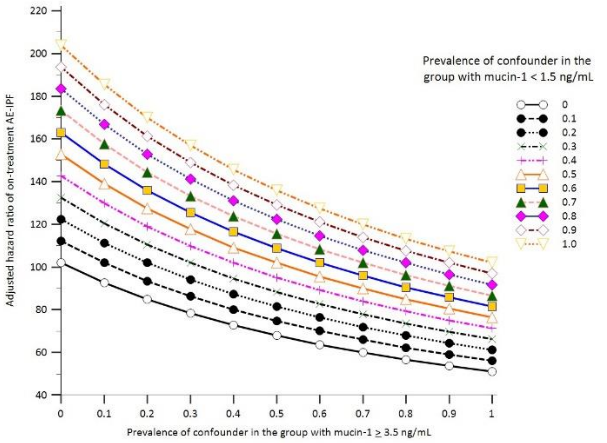

Supplemental Figure S1d

Supplemental Figure S1. Explanation:

Results of the sensitivity analyses as presented graphically above show that regardless of the prevalence of the potentially unidentified confounder: (a) baseline plasma mucin-1 level ≥ 2.5 ng/mL is still a significant predictor of on-treatment AE-IPF; the incremental trend in adjusted hazard ratios of AE-IPF persists across subgroups of increasing levels of plasma mucin-1 (b) ≥ 1.5 but < 2.5; (c) ≥ 2.5 but < 3.5; (d) ≥ 3.5; ng/mL. For example, as in Supplemental Figure S1a, when hypothetically all the patients with baseline mucin-1 < 2.5 ng/mL have the potentially unidentified confounder (and the prevalence of this confounder was therefore 1.0, as represented by the top orange broken line with hallow-triangular markers), but none of the patients with baseline mucin-1 ≥ 2.5 ng/mL have this unidentified confounder (and thus the prevalence of this confounder was 0), then having baseline mucin-1 ≥ 2.5 ng/mL would still be a significant predictor of AE-IPF (with the adjusted hazard ratio = 28.15). Sensitivity analyses were performed using R (Version 3.6.3) and the packages ***survival*** and ***obsSens***; the graphs were plotted using MedCal (Version 20.118).

Supplemental Figure S1. Abbreviation: AE-IPF, acute exacerbation of idiopathic pulmonary fibrosis.

Supplemental Figure S2. Sensitivity analyses of the multi-variable Cox proportional hazards regression models for ***early*** severe adverse outcomes (SAO).

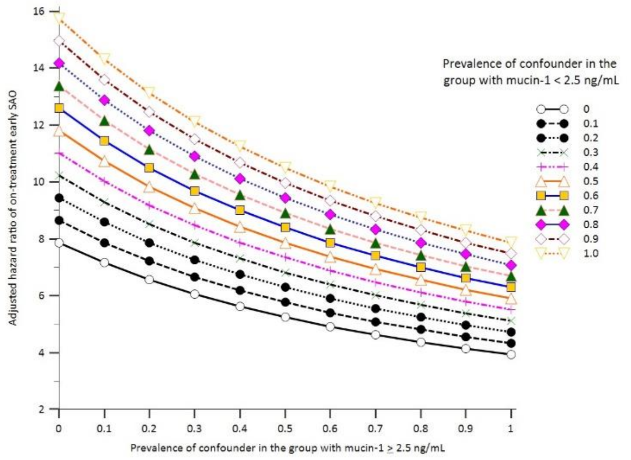

Supplemental Figure S2a

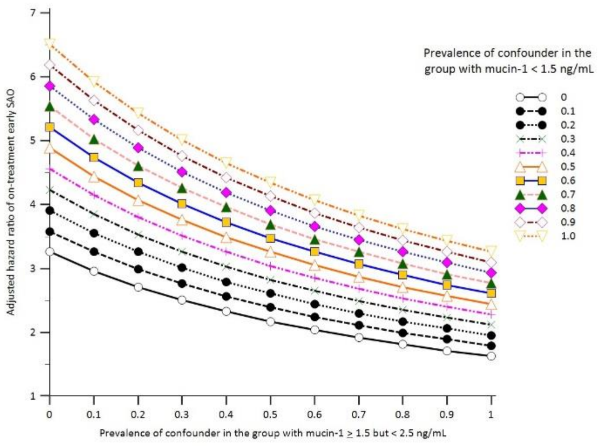

Supplemental Figure S2b

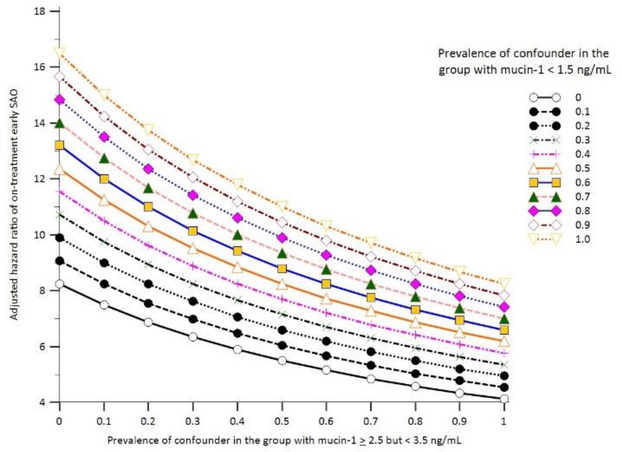

Supplemental Figure S2c

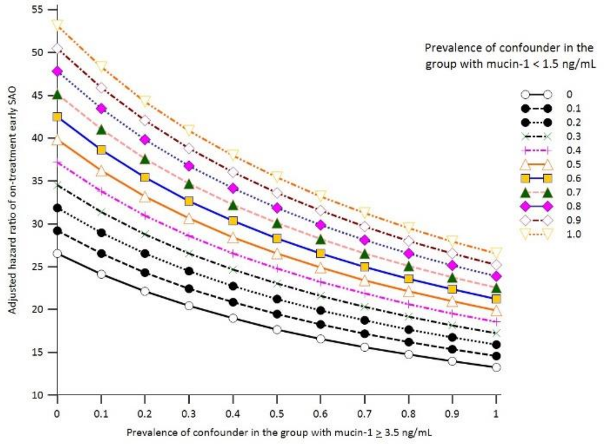

Supplemental Figure S2d

Supplemental Figure S2. Explanation:

Results of the sensitivity analyses as presented graphically above show that regardless of the prevalence of the potentially unidentified confounder: (**a**) baseline plasma mucin-1 level ≥ 2.5 ng/mL is still a significant predictor of on-treatment early SAO; the incremental trend in adjusted hazard ratios of early SAO persists across subgroups of increasing levels of plasma mucin-1 (**b**) ≥ 1.5 but < 2.5; (**c**) ≥ 2.5 but < 3.5; (**d**) ≥ 3.5; ng/mL. For example, as in Supplemental Figure S2a, when hypothetically all the patients with baseline mucin-1 < 2.5 ng/mL have the potentially unidentified confounder (and the prevalence of this confounder was therefore 1.0, as represented by the top orange broken line with hallow-triangular markers), but none of the patients with baseline mucin-1 ≥ 2.5 ng/mL have this unidentified confounder (and thus the prevalence of this confounder was 0), then having baseline mucin-1 ≥ 2.5 ng/mL would still be a significant predictor of SAO (with the adjusted hazard ratio = 15.74). Sensitivity analyses were performed using R (Version 3.6.3) and the packages ***survival*** and ***obsSens***; the graphs were plotted using MedCal (Version 20.118).

Supplemental Figure S2. Abbreviation: SAO, severe adverse outcomes (including on-treatment lung transplantation and all-cause mortality).

Supplemental Figure S3. Sensitivity analyses of the multi-variable Cox proportional hazards regression models for the risk of severe adverse outcomes (SAO) anytime during the follow-up period.

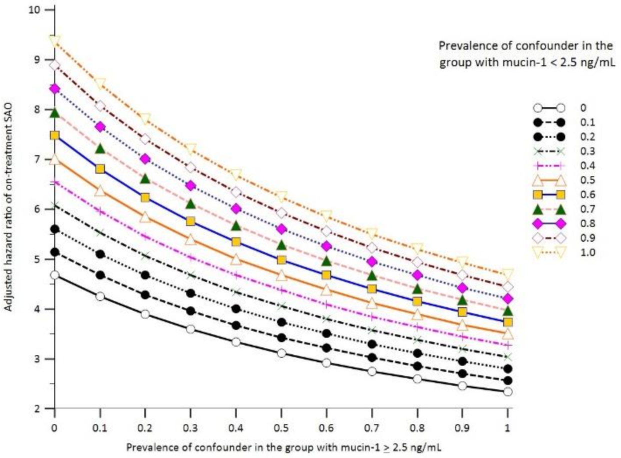

Supplemental Figure S3a

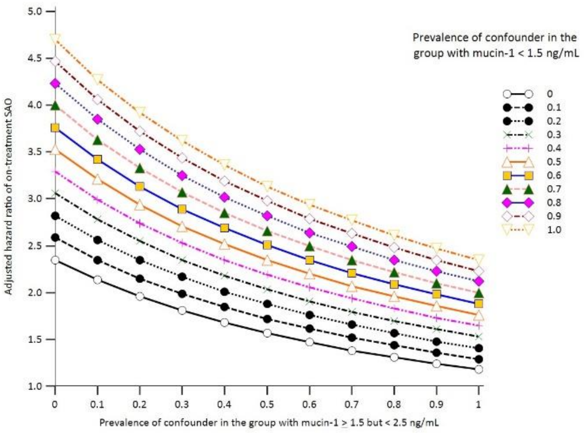

Supplemental Figure S3b

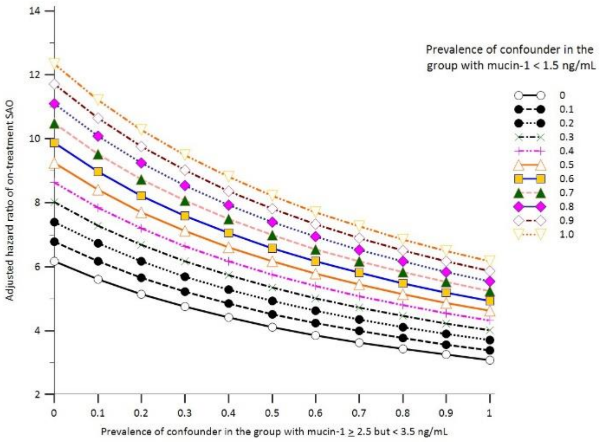

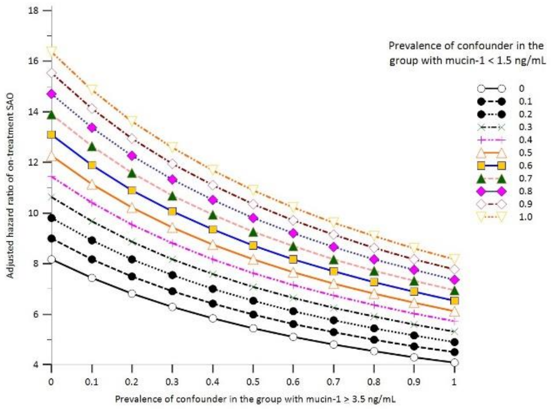

Supplemental Figure S3c

Supplemental Figure S3d

Supplemental Figure S3. Explanation:

Results of the sensitivity analyses as presented graphically above show that regardless of the prevalence of the potentially unidentified confounder: (**a**) baseline plasma mucin-1 level ≥ 2.5 ng/mL is still a significant predictor of on-treatment SAO; the incremental trend in adjusted hazard ratios of SAO persists across subgroups of increasing levels of plasma mucin-1 (**b**) ≥ 1.5 but < 2.5; (**c**) ≥ 2.5 but < 3.5; (**d**) ≥ 3.5; ng/mL. For example, as in Supplemental Figure S2a, when hypothetically all the patients with baseline mucin-1 < 2.5 ng/mL have the potentially unidentified confounder (and the prevalence of this confounder was therefore 1.0, as represented by the top orange broken line with hallow-triangular markers), but none of the patients with baseline mucin-1 ≥ 2.5 ng/mL have this unidentified confounder (and thus the prevalence of this confounder was 0), then having baseline mucin-1 ≥ 2.5 ng/mL would still be a significant predictor of SAO (with the adjusted hazard ratio = 9.36). Sensitivity analyses were performed using R (Version 3.6.3) and the packages ***survival*** and ***obsSens***; the graphs were plotted using MedCal (Version 20.118).

Supplemental Figure S3. Abbreviation: SAO, severe adverse outcomes (including on-treatment lung transplantation and all-cause mortality).

Appendix S1. Formulae for the calculation of rates of changes in pulmonary functional parameters.

1. Calculation of 24-week rate of change:

   (1) $[(FVC_B - FVC_A)$ / (time between test A and test B in weeks)] x 24

   (2) $[(D_{LCO\ B} - D_{LCO\ A})$ / (time between test A and test B in weeks)] x 24

2. Calculation of annualized (52-week) rate of change:

   (1) $[(FVC_B - FVC_A)$ / (time between test A and test B in weeks)] x 52

   (2) $[(D_{LCO\ B} - D_{LCO\ A})$ / (time between test A and test B in weeks)] x 52

Note: FVC represents forced vital capacity and D_{LCO} represents diffusion capacity for carbon monoxide. The subscript "A" indicates measurements that were closest in time before the initiation of pirfenidone treatment, and the subscript "B" indicates measurements that were closest in time before or within 28 days after the last dose of pirfenidone (for patients who prematurely discontinued the treatment) or September 30, 2023 (for patients who continued the treatment).
